# Supplementary material for: Modeling glioblastoma heterogeneity as a dynamic network of cell states
Source: Mol Syst Biol. 2021 Sep 16;17(9):e10105. doi: 10.15252/msb.202010105 (PMC8444284; doi:10.15252/msb.202010105)
Supplement: Supplementary file 6 — Source Data for Figure 5 [file MSB-17-e10105-s004.zip › Figure5A_sourcedata/GSEA_3017/nosigdb_stateE.GseaPreranked.1622795031093/NB_KI_CLUSTER5.html]

Details for gene set NB\_KI\_CLUSTER5[GSEA]

|  || Dataset | state63017 |
| Phenotype | NoPhenotypeAvailable |
| Upregulated in class | na\_neg |
| GeneSet | NB\_KI\_CLUSTER5 |
| Enrichment Score (ES) | -0.6791405 |
| Normalized Enrichment Score (NES) | -3.4579835 |
| Nominal p-value | 0.0 |
| FDR q-value | 0.0 |
| FWER p-Value | 0.0 |
Table: GSEA Results Summary

  

Fig 1: Enrichment plot: NB\_KI\_CLUSTER5      
 Profile of the Running ES Score & Positions of GeneSet Members on the Rank Ordered List

  

| PROBE | GENE SYMBOL | GENE\_TITLE | RANK IN GENE LIST | RANK METRIC SCORE | RUNNING ES | CORE ENRICHMENT || 1 | RAN |  |  | 298 | -0.257 | -0.3394 | No |
| 2 | MCM5 |  |  | 337 | -0.265 | -0.3785 | No |
| 3 | NASP |  |  | 410 | -0.285 | -0.4564 | No |
| 4 | DNMT1 |  |  | 466 | -0.304 | -0.5144 | No |
| 5 | GGH |  |  | 504 | -0.314 | -0.5514 | No |
| 6 | RHEB |  |  | 528 | -0.323 | -0.5721 | No |
| 7 | MCM3 |  |  | 560 | -0.340 | -0.6017 | No |
| 8 | TCF19 |  |  | 569 | -0.342 | -0.6047 | No |
| 9 | ZWILCH |  |  | 604 | -0.360 | -0.6374 | No |
| 10 | DDX11 |  |  | 622 | -0.370 | -0.6503 | No |
| 11 | CDKN2D |  |  | 638 | -0.381 | -0.6607 | No |
| 12 | SHCBP1 |  |  | 655 | -0.398 | -0.6719 | Yes |
| 13 | GINS2 |  |  | 658 | -0.399 | -0.6669 | Yes |
| 14 | CDT1 |  |  | 665 | -0.406 | -0.6665 | Yes |
| 15 | NUDT1 |  |  | 674 | -0.413 | -0.6682 | Yes |
| 16 | H2AFV |  |  | 684 | -0.423 | -0.6709 | Yes |
| 17 | FANCA |  |  | 691 | -0.425 | -0.6701 | Yes |
| 18 | DIAPH3 |  |  | 696 | -0.427 | -0.6669 | Yes |
| 19 | RANBP1 |  |  | 697 | -0.427 | -0.6591 | Yes |
| 20 | ANP32B |  |  | 699 | -0.431 | -0.6524 | Yes |
| 21 | CHEK1 |  |  | 704 | -0.433 | -0.6492 | Yes |
| 22 | TEX30 |  |  | 709 | -0.436 | -0.6458 | Yes |
| 23 | MZT1 |  |  | 711 | -0.437 | -0.6390 | Yes |
| 24 | LBR |  |  | 739 | -0.463 | -0.6618 | Yes |
| 25 | NUCKS1 |  |  | 744 | -0.471 | -0.6578 | Yes |
| 26 | LIG1 |  |  | 752 | -0.485 | -0.6571 | Yes |
| 27 | PRR11 |  |  | 755 | -0.488 | -0.6505 | Yes |
| 28 | SPDL1 |  |  | 758 | -0.491 | -0.6438 | Yes |
| 29 | TRIP13 |  |  | 760 | -0.498 | -0.6359 | Yes |
| 30 | MXD3 |  |  | 763 | -0.502 | -0.6291 | Yes |
| 31 | GMNN |  |  | 766 | -0.510 | -0.6221 | Yes |
| 32 | NCAPD2 |  |  | 784 | -0.540 | -0.6319 | Yes |
| 33 | CEP152 |  |  | 785 | -0.542 | -0.6220 | Yes |
| 34 | HMGN2 |  |  | 795 | -0.564 | -0.6221 | Yes |
| 35 | RACGAP1 |  |  | 796 | -0.566 | -0.6118 | Yes |
| 36 | HMGB1 |  |  | 797 | -0.567 | -0.6015 | Yes |
| 37 | TUBB4B |  |  | 804 | -0.573 | -0.5980 | Yes |
| 38 | PLK1 |  |  | 809 | -0.586 | -0.5919 | Yes |
| 39 | CHAF1A |  |  | 810 | -0.588 | -0.5812 | Yes |
| 40 | DEK |  |  | 812 | -0.591 | -0.5716 | Yes |
| 41 | MCM7 |  |  | 817 | -0.599 | -0.5653 | Yes |
| 42 | HELLS |  |  | 830 | -0.635 | -0.5676 | Yes |
| 43 | PCNA |  |  | 832 | -0.640 | -0.5571 | Yes |
| 44 | BUB3 |  |  | 834 | -0.641 | -0.5466 | Yes |
| 45 | CENPW |  |  | 835 | -0.643 | -0.5349 | Yes |
| 46 | ANP32E |  |  | 841 | -0.676 | -0.5283 | Yes |
| 47 | BIRC5 |  |  | 843 | -0.679 | -0.5171 | Yes |
| 48 | CENPM |  |  | 844 | -0.682 | -0.5047 | Yes |
| 49 | BRCA1 |  |  | 846 | -0.691 | -0.4933 | Yes |
| 50 | ZWINT |  |  | 847 | -0.692 | -0.4807 | Yes |
| 51 | DHFR |  |  | 851 | -0.701 | -0.4714 | Yes |
| 52 | CIP2A |  |  | 853 | -0.712 | -0.4595 | Yes |
| 53 | ECT2 |  |  | 854 | -0.717 | -0.4465 | Yes |
| 54 | CENPK |  |  | 857 | -0.722 | -0.4356 | Yes |
| 55 | FBXO5 |  |  | 864 | -0.742 | -0.4290 | Yes |
| 56 | KIF20B |  |  | 876 | -0.779 | -0.4275 | Yes |
| 57 | CKAP2 |  |  | 878 | -0.781 | -0.4145 | Yes |
| 58 | PTTG1 |  |  | 880 | -0.791 | -0.4012 | Yes |
| 59 | EZH2 |  |  | 886 | -0.810 | -0.3922 | Yes |
| 60 | UBE2S |  |  | 887 | -0.815 | -0.3774 | Yes |
| 61 | H2AFZ |  |  | 893 | -0.849 | -0.3677 | Yes |
| 62 | KPNA2 |  |  | 895 | -0.858 | -0.3532 | Yes |
| 63 | DBF4 |  |  | 896 | -0.864 | -0.3375 | Yes |
| 64 | SMC2 |  |  | 897 | -0.867 | -0.3217 | Yes |
| 65 | SGO2 |  |  | 898 | -0.869 | -0.3058 | Yes |
| 66 | SMC4 |  |  | 902 | -0.886 | -0.2932 | Yes |
| 67 | DUT |  |  | 903 | -0.887 | -0.2770 | Yes |
| 68 | MIS18BP1 |  |  | 904 | -0.889 | -0.2608 | Yes |
| 69 | CKS1B |  |  | 909 | -0.912 | -0.2488 | Yes |
| 70 | CKS2 |  |  | 911 | -0.922 | -0.2332 | Yes |
| 71 | CLSPN |  |  | 916 | -0.964 | -0.2203 | Yes |
| 72 | PRC1 |  |  | 919 | -0.980 | -0.2047 | Yes |
| 73 | UBE2T |  |  | 922 | -0.994 | -0.1889 | Yes |
| 74 | HIST1H1E |  |  | 925 | -1.049 | -0.1721 | Yes |
| 75 | ATAD2 |  |  | 933 | -1.162 | -0.1590 | Yes |
| 76 | HIST2H2AC |  |  | 934 | -1.162 | -0.1379 | Yes |
| 77 | HIST1H1C |  |  | 936 | -1.220 | -0.1168 | Yes |
| 78 | HMGB2 |  |  | 937 | -1.238 | -0.0943 | Yes |
| 79 | CDK1 |  |  | 938 | -1.248 | -0.0715 | Yes |
| 80 | PCLAF |  |  | 943 | -1.554 | -0.0478 | Yes |
| 81 | HIST1H4C |  |  | 946 | -2.752 | 0.0000 | Yes |
Table: GSEA details [plain text format]

  

Fig 2: NB\_KI\_CLUSTER5: Random ES distribution      
 Gene set null distribution of ES for **NB\_KI\_CLUSTER5**
